# Editorial: Evaluating efficacy and outcomes in neonatal HIE treatment: A global perspective

**DOI:** 10.3389/fped.2026.1939776

**Published:** 2026-08-25

**Authors:** Alan R. Horn, Helga Naburi, Hemmen Sabir, Kondwani Kawaza, Shakti Pillay

**Affiliations:** 1 Department of Paediatrics and Child Health, Division of Neonatology, University of Cape Town, Cape Town, South Africa; 2 Department of Paediatrics and Child Health, Muhimbili University of Health and Allied Sciences, Dar es Salaam, Tanzania; 3 Experimental Neonatology, University Hospitals Bonn, Bonn, Germany; 4 Department of Paediatrics and Child Health, Kamuzu University of Health Sciences, Blantyre, Malawi

**Keywords:** hypothermia—induced, hypoxia ischaemia—brain, low- and middle-income countries, neonatal encephalopathy, neonate, neurodevelopmental, neuroprotection, outcomes

## Introduction

Hypoxic–ischaemic encephalopathy (HIE) is a leading cause of acquired neonatal brain injury, with a disproportionate burden in low- and middle-income countries (LMICs) (1, 2). Although therapeutic hypothermia (TH) has transformed the management of moderate and severe HIE over the past two decades, outcomes from large randomised trials show that 46%–50% of cooled neonates, in multiple settings, either die or survive with significant neurological impairment (3, 4). The effectiveness of TH in middle-income countries appears to be comparable to high-income countries, but data tend to be confounded by variable treatment and assessment methodologies, and high loss to long-term follow-up (5, 6). The research challenge is no longer simply whether neuroprotection works, but also which infants benefit, how treatment response can be recognised early, how supportive care can be optimised, and which additional therapies further improve outcomes, particularly in LMIC settings (7). This Research Topic presents a collection of papers which explore these questions.

## The global context and evolving landscape

A recurring message across the collection is the importance of understanding HIE within its global context. Shu et al. provide a bibliometric analysis of over one thousand publications on TH and neonatal HIE. They document the rapid expansion of international collaboration and the shift in research priorities from the efficacy of TH towards understanding pathophysiology and inflammatory pathways and identifying early biomarkers—developing the potential for mechanism-driven neurocritical neonatal care. The global perspective, is further developed in the retrospective analysis of the Global Burden of Disease (GBD) dataset from 1990 to 2021, by Du et al. They demonstrate a decrease in the incidence of neonatal encephalopathy due to birth asphyxia and trauma, with associated decreased mortality and disability-adjusted life years, but persisting socioeconomic inequities in access to perinatal care and a growing population of survivors with disability. Notwithstanding the limited GBD data from low-resource settings, their findings highlight the need for neuroprotective therapies to be scalable, affordable and implementable across diverse healthcare settings. One such widely available potential candidate is caffeine—based on high-dose studies to prevent intermittent hypoxia in preterm and late-preterm neonates (8–12), pre-clinical studies to treat HIE (13–19), and a completed phase 1 human safety trial (20, 21).

## Early diagnosis and prognostication

Several papers in the research topic focus on early diagnosis and prognostication. A parental survey by Abdelrahman et al. demonstrated that a third of neonates who were considered healthy at birth, but with umbilical cord pH values of 7.0–7.15, showed developmental delay at age two years. Their findings suggest that the risk of long-term developmental impairment may be underestimated by relying solely on current HIE definitions. They also support the use of an early pH of ≤7.15 as a criteria for neurological assessment to determine eligibility for neuroprotection treatment, as applied in several TH trials (3).

The theme of early recognition of potential injury is further developed by several papers in the research topic examining objective biomarkers and prediction models. Li et al. demonstrate that dynamic neutrophil ratio trajectories following hypoxic-ischaemic injury were associated with short-term neurological outcomes. Their work supports the potential value of immune-inflammatory profiling in early risk-stratification and individualized care. Van der Donk et al. propose a prognostic score that combines eight clinical variables, which were routinely collected in their centre, to predict adverse outcomes at 1 year. Although their model still requires internal and external validation, they illustrate the movement towards multiple predictors integrating clinical, laboratory and physiological data. This trend is further illustrated by the findings of the systematic review of outcome predictors following neonatal encephalopathy in LMICS, by. Although data synthesis and comparability were limited by substantial heterogeneity between study populations, the reported candidate biomarkers included cytokines, neuron-specific enolase, glial fibrillary acidic protein and markers of DNA damage. Magnetic resonance imaging (MRI) showed high specificity, while early severe electroencephalography (EEG) abnormalities demonstrated the highest sensitivity and specificity. Similarly, background EEG abnormalities were strongly associated with neurological outcome, in the large study of continuous video EEG recordings during TH for HIE, by Woodward et al., in keeping with studies of amplitude integrated EEG (aEEG) during TH for HIE (22, 23). In contrast, cumulative ictal burden and acute antiseizure medication exposure did not predict outcome in the study by Woodward et al.

The ability of early assessment after discharge is particularly relevant in settings where loss to long-term follow-up is high. Kali et al. showed the limitations of comparing General Movements Assessment (GMA), Motor Optimality Score-Revised (MOS-R) and the Hammersmith Infant Neurological Examination (HINE) at 12–15 weeks, with Bayley-III assessments at 12 and/or 18 months, in neonates treated with TH for HIE in South Africa. Combined GMA, MOS-R and HINE had high sensitivity and negative predictive value but low specificity and positive predictive value. The findings underscore the need for local validation of neurological assessments, which has been done for the Hammersmith Neonatal Neurological Examination (HNNE) (24).

### Optimising therapeutic hypothermia and supportive care

Therapeutic hypothermia extends beyond temperature management alone. Optimal and standardised supportive care probably contributes substantially to outcome and facilitates more robust interpretation of the effect of neuroprotective interventions (25). Samaai et al. challenge historical concerns regarding enteral feeding during cooling and provide practical guidance. They showed that a standardised progressive early enteral feeding strategy during therapeutic hypothermia was well tolerated, with low mortality and no cases of necrotising enterocolitis. Feed intolerance was associated with more severe encephalopathy and persistent aEEG suppression at 48 h. The small retrospective case-controlled study by Huang et al. demonstrated short-term benefits of servo-controlled active cooling during neonatal transport, in their high-resource setting. Their work suggests that treatment is optimal when accompanied by early recognition, adequate transport infrastructure, and reliable temperature control.

## Conclusion

These papers include epidemiology, prognostication, biomarkers, neurophysiology, developmental assessment, nutritional management, and research trends, reflecting the increasingly multidisciplinary nature of neonatal neuroprotection. Ultimately, the future of effective neonatal neuroprotection will depend on recognising the biological heterogeneity of HIE and delivering the right intervention to the right infant at the right time.
